# Identification of RT-PCR-Negative Asymptomatic COVID-19 Patients via Serological Testing

**DOI:** 10.3389/fpubh.2020.00267

**Published:** 2020-06-05

**Authors:** Jinru Wu, Xinyi Liu, Dan Zhou, Guangqian Qiu, Miao Dai, Qingting Yang, Zhonghui Pan, Ning Zhou, Pa Wu

**Affiliations:** ^1^College of Life Science, Hunan Normal University, Changsha, China; ^2^Loudi Center for Disease Control and Prevention, Loudi, China; ^3^Xinhua Center for Disease Control and Prevention, Xinhua, China; ^4^Department of Infectious Diseases, The Second Xiangya Hospital, Central South University, Changsha, China

**Keywords:** COVID-19, asymptomatic, serological test, SARS-CoV-2, reverse transcription polymerase chain reaction

## Abstract

Asymptomatic individuals with coronavirus disease (COVID-19) have been identified via nucleic acid testing for severe acute respiratory syndrome coronavirus 2 (SARS-CoV-2); however, the epidemiologic characteristics and viral shedding pattern of asymptomatic patients remain largely unknown. In this study, serological testing was applied when identifying nine asymptomatic cases of COVID-19 who showed persistent negative RT-PCR test results for SARS-CoV-2 nucleic acid and no symptoms of COVID-19. Two asymptomatic cases were presumed to be index patients who had cleared the virus when their close contacts developed symptoms of COVID-19. Three of the asymptomatic cases were local individuals who spontaneously recovered before their presumed index patients developed symptoms of COVID-19. This report presents the epidemiologic and clinical characteristics of asymptomatic individuals with SARS-CoV-2 infection that were undetected on RT-PCR tests in previous epidemiologic investigations probably due to the transient viral shedding duration.

## Introduction

The coronavirus disease (COVID-19) has spread globally, mainly via person-to-person transmission, and poses a major public health concern (1). The epidemiologic and clinical characteristics of symptomatic COVID-19 patients have been increasingly reported in recent research (2), whereas the asymptomatic proportion of severe acute respiratory syndrome coronavirus 2 (SARS-CoV-2)-infected individuals remains largely uncharacterized.

Symptomatic patients are detected because they seek medical attention, but asymptomatic individuals with SARS-CoV-2 infection are identified via the reverse transcription polymerase chain reaction (RT-PCR) test for SARS-CoV-2 (3). A previous study investigated a familial cluster of COVID-19 cases that included an asymptomatic 10-year-old boy who had radiological ground-glass lung opacities and tested positive on the RT-PCR test for SARS-CoV-2 (1). A case report identified a 61-year-old asymptomatic patient with abnormal CT images and positive RT-PCR test results for SARS-CoV-2 that persisted for 23 days in the absence of obvious clinical symptoms (4). While some asymptomatic infected individuals remain asymptomatic over a long period, a proportion of asymptomatic COVID-19 patients were identified at the presymptomatic stage. Through epidemiological investigation, a study identified 24 SARS-CoV-2 positive cases on RT-PCR comprising five cases that subsequently developed COVID-19 symptoms and 19 that remained asymptomatic (5). In addition, a study screened pregnant women who presented at hospitals and identified 43 COVID-19 patients via nucleic acid tests, including four patients who remained afebrile and asymptomatic throughout their delivery hospitalization and postpartum course (6). The abovementioned studies identified asymptomatic COVID-19 patients via RT-PCR tests, but the viral shedding pattern and epidemiologic characteristics of COVID-19 remain poorly understood.

Asymptomatic patients can easily be overlooked in epidemic prevention (7, 8). Based on data extracted from China's Infectious Disease Information System, 889 asymptomatic cases were identified among the 72,314 patient records, which accounted for only 1.2% of the total patients (9). Recent RT-PCR tests in China that primarily provided for passengers arriving from abroad revealed that 78% of the cases of new infection were asymptomatic when the tests were conducted (10). The proportion of asymptomatic COVID-19 patients on the Diamond Princess cruise ship was estimated to be 50.5%, and the proportion among the evacuated Japanese citizens was estimated to be 30.8% (3, 11). In addition, a study suggested that at least 59% of the infected cases went undetected in Wuhan, which potentially includes asymptomatic and mildly symptomatic cases (8, 12). In this study, we enrolled 38 patients with close contact with COVID-19 who exhibited persistently negative RT-PCR results for SARS-CoV-2 and aimed to evaluate their antibody for SARS-CoV-2 via serological tests. To the best of our knowledge, this report is among the first reports on tracing of the index patients via serology testing.

## Case Report

**Figure 1:**
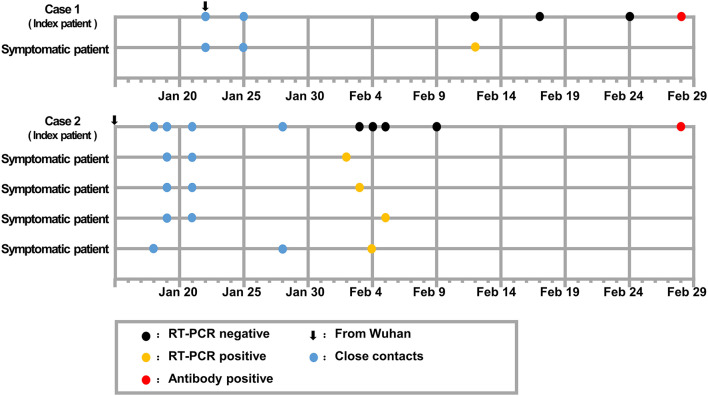
Timeline of epidemiologic and clinic events in asymptomatic carrier transmission of COVID-19.

**Figure 2:**
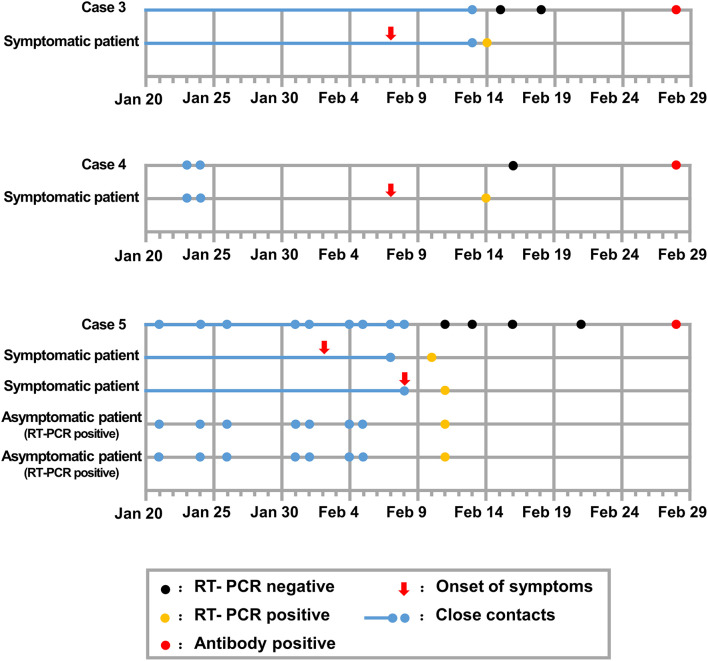
Chronology of epidemiologic and clinic events of asymptomatic local cases presumably infected by their close contacts.

**Figure 3:**
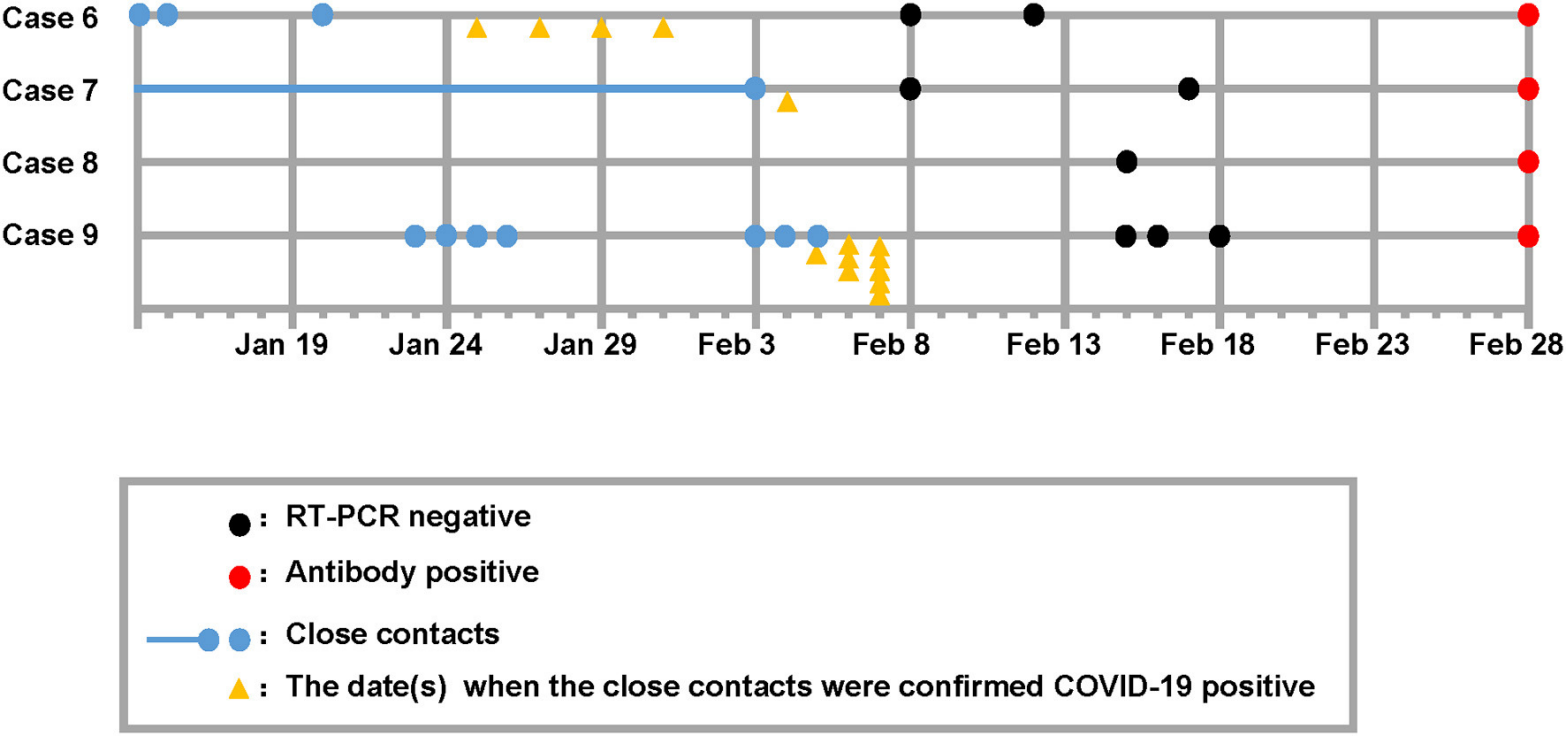
Timeline of epidemiologic and clinic events of asymptomatic patients who had undefined role in disease transmission.

Among the 38 study participants enrolled in this study, the median age of the patients was 47.5 years (interquartile range 26.5–59.25), and 11 (28.9%) were female. Of the 38 close contacts who were tested, nine cases were found positive for anti-SARS-CoV-2 antibody. The positive serologic test results were reported with consistent results from Wondfo and Bioscience Biotechnology Co. Ltd., both of which have been approved by the Chinese National Drug Administration. Colloidal gold immunochromatography reagent from Wondfo Biotechnology Co. Ltd. and chemiluminescence reagent for immunoglobulin G from Bioscience Biotechnology Co. Ltd. were used to detect SARS-CoV-2 specific antibodies according to the manufacturer's instructions. Of the nine asymptomatic cases, two cases were presumed to be index patients of local cases (Figure 1), three cases were local patients who spontaneously recovered before their presumed index patients were found positive on SARS-CoV-2 nucleic acid testing (Figure 2), and four cases were close contacts of COVID-19 patients and had undefined roles in disease transmission (Figure 3).

Case 1 was a 39-year-old woman who lived in Wuhan and drove to Loudi on January 22, 2020, to visit a relative, who developed fever and cough on February 9, 2020, and tested positive on RT-PCR for SARS-CoV-2 on February 12, 2020 (Figure 1). Case 1 showed negative RT-PCR test results for SARS-CoV-2 on nasopharyngeal swab samples collected on February 12, 17, and 24 and exhibited no fever, cough, dyspnea, headache, fatigue, gastrointestinal, or other COVID-19 symptoms, and she did not receive any therapeutic intervention over this period. A blood sample collected on February 28, 2020, showed that she tested positive on a serological test for SARS-CoV-2 (Figure 1). Case 1 was presumed to have viral shedding during January 22 and January 25, 2020; she spontaneously recovered before February 12, 2020 and had no COVID-19-related symptoms during the following 2 months.

Case 2, a 52-year-old man, lived in Wuhan and took the train to Loudi on January 15, 2020. Four close contacts of Case 2 were later diagnosed with COVID-19 (Figure 1). Specifically, one close contact had never been in contact with the other three close contacts. Case 2 presented with occasional cough but denied any fever, dyspnea, headache, fatigue, or gastrointestinal symptoms over the period. As Case 2 had an occasional cough and four of his close contacts were confirmed to have COVID-19, Case 2 underwent medical examination from February 3 to 5. Chest computed tomography (CT) scanning images recorded on February 4 and 7 showed exudative lesions in both lungs. However, the RT-PCR tests for SARS-CoV-2 that were repeated four times with nasopharyngeal swab samples were all negative (Figure 1), whereas the anti-*Mycoplasma pneumoniae* immunoglobulin M (IgM) and IgG antibody tests on February 7 were positive. During the 2-week home quarantine without specific therapeutic intervention, Case 2 reported that his cough had resolved. No characteristic symptom of COVID-19 was reported thereafter; however, a blood sample collected on February 28, 2020, tested positive for anti-SARS-CoV-2 antibody (Figure 1), although Case 2 had no COVID-19-related symptoms or complications during the following 2 months.

Cases 3–5 were local residents without travel histories in the past 3 months and were infected by the presumed index patients from the previous epidemiologic investigation. These patients presumably recovered spontaneously before their index patients were confirmed positive for SARS-CoV-2 (Figure 2). Cases 6–9 had been to Wuhan or had traveled since the COVID-19 outbreak, and they thus had an undefined role in disease transmission (Figure 3). Cases 3–9 had persistently negative RT-PCR test results for SARS-CoV-2 without COVID-19 symptoms but tested positive on serological tests for SARS-CoV-2. The cases denied previous exposure to SARS-CoV-2 and received no therapeutic intervention or complained of COVID-19-related symptoms or complications during the following 2 months.

## Discussion

Nine additional asymptomatic patients of COVID-19 were identified via serological tests in 38 close contacts of COVID-19 patients who had persistently negative RT-PCR test results for SARS-CoV-2. Local COVID-19 cases are rare in Loudi District, which has a total of 76 symptomatic patients and 26 asymptomatic patients who were identified via RT-PCR test for SARS-CoV-2.

Underestimation of asymptomatic infections of COVID-19 has been suggested, as an increasing number of infected people have not traveled to epidemic hotspots or been linked to known COVID-19 patients (8). Applying serological tests, Singapore identified the source of a cluster of 23 COVID-19 patients who tested negative on RT-PCR for SARS-CoV-2 (13). In this study, we report nine asymptomatic patients identified via serological test in addition to 26 asymptomatic patients who were previously identified by the RT-PCR test. The nine asymptomatic patients had multiple negative RT-PCR test results, with the first RT-PCR test of these asymptomatic patients conducted almost immediately after their close contacts were identified as COVID-19 patients. Thus, a proportion of asymptomatic patients might have short viral shedding duration or may have viral nucleic acid loads that are undetectable on RT-PCR. According to a previous study, the interval from the first day of positive RT-PCR tests to the first day of continuous negative tests for the asymptomatic patient ranged from 1 to 21 days, with five asymptomatic patients having persistently negative RT-PCR test results 1 day after the date of diagnosis (5). The results indicated the potential for the underestimation of the proportion of asymptomatic patients based on RT-PCR tests, which possibly identifies only those with longer viral shedding period. These results suggested that serological tests could serve as a more reliable method to estimate the asymptomatic proportion of COVID-19 patients.

Studies have suggested that symptomatic patients of COVID-19 have higher transmissibility within 5 days of symptom onset than later on, and infectivity might peak on or before symptom onset (14, 15). However, the epidemiologic characteristics of asymptomatic patients remain unclear. A previous study has identified COVID-19 transmission caused by an asymptomatic carrier who had normal chest CT findings (16). In addition, the viral load detected in the asymptomatic patient was similar to that in the symptomatic patients, suggesting a similar transmission potential (17). Herein, we identified COVID-19 transmission caused by two asymptomatic index patients who cleared the virus whereas their local relatives developed symptoms of COVID-19.

Serological test results of the 9 asymptomatic patients who had repeated negative RT-PCR test results for SARS-CoV-2 suggest that a proportion of the individuals infected with SARS-CoV-2 can recover without treatment, indicating that some individuals may have highly efficient neutralizing antibodies. Admittedly, this research was limited to a small cohort with nasopharyngeal swab samples of the asymptomatic cases collected after their close contacts were confirmed to be SARS-CoV-2 positive on RT-PCR. Further studies with a large cohort are needed to elucidate the viral shedding pattern and transmission characteristics of asymptomatic cases with SARS-CoV-2 infection.

## Ethics Statement

This study was approved by the Hunan Normal University institutional review board. Written informed consent was obtained from the participants for the publication of the case report.

## Author Contributions

PW and JW conceived and designed the experiments. XL, JW, GQ, MD, QY, DZ, and ZP contributed to the acquisition, analysis, or interpretation of data. PW and NZ drafted and revised the manuscript. All authors critically reviewed the manuscript.

## Conflict of Interest

The authors declare that the research was conducted in the absence of any commercial or financial relationships that could be construed as a potential conflict of interest.
